# A robust and reproducible animal serum-free culture method for clinical-grade bone marrow-derived mesenchymal stromal cells

**DOI:** 10.1007/s10616-014-9841-x

**Published:** 2015-03-17

**Authors:** Anita Laitinen, Sofia Oja, Lotta Kilpinen, Tanja Kaartinen, Johanna Möller, Saara Laitinen, Matti Korhonen, Johanna Nystedt

**Affiliations:** Research and Cell Therapy Services, Finnish Red Cross Blood Service, Kivihaantie 7, 00310 Helsinki, Finland

**Keywords:** Mesenchymal stromal cell (MSC), Low oxygen, Platelet lysate (PL), FBS, HLA-DR

## Abstract

Efficient xenofree expansion methods to replace fetal bovine serum (FBS)-based culture methods are strongly encouraged by the regulators and are needed to facilitate the adoption of mesenchymal stromal cell (MSC)-based therapies. In the current study we established a clinically-compliant and reproducible animal serum-free culture protocol for bone marrow-(BM-) MSCs based on an optimized platelet-derived supplement. Our study compared two different platelet-derived supplements, platelet lysate PL1 versus PL2, produced by two different methods and lysed with different amounts of freeze–thaw cycles. Our study also explored the effect of a low oxygen concentration on BM-MSCs. FBS-supplemented BM-MSC culture served as control. Growth kinetics, differentiation and immunomodulatory potential, morphology, karyotype and immunophenotype was analysed. Growth kinetics in long-term culture was also studied. Based on the initial results, we chose to further process develop the PL1-supplemented culture protocol at 20 % oxygen. The results from 11 individual BM-MSC batches expanded in the chosen condition were consistent, yielding 6.60 × 10^9^ ± 4.74 × 10^9^ cells from only 20 ml of bone marrow. The cells suppressed T-cell proliferation, displayed normal karyotype and typical MSC differentiation potential and phenotype. The BM-MSCs were, however, consistently HLA-DR positive when cultured in platelet lysate (7.5–66.1 %). We additionally show that culture media antibiotics and sterile filtration of the platelet lysate can be successfully omitted. We present a robust and reproducible clinically-compliant culture method for BM-MSCs based on platelet lysate, which enables high quantities of HLA-DR positive MSCs at a low passage number (p2) and suitable for clinical use.

## Introduction

Mesenchymal stromal cells (MSCs) are multipotent non-hematopoietic cells that are commonly isolated from bone marrow (BM) or adipose tissue. In the BM, these cells comprise only a small population of cells, 0.001–0.01 % (Pittenger et al. 1999), however they can be isolated and expanded for several passages in vitro. MSCs are able to differentiate to cell types of mesodermal origin such as adipocytes, chondrocytes and osteoblasts (Pittenger et al. 1999), and have thus generated interest in their potential application in tissue regenerative therapies (Dimarino et al. 2013; Sensebe and Bourin 2009). MSCs also potently suppress T-cell mediated rejection reactions and ameliorate clinical graft-versus-host reactions (Aggarwal and Pittenger 2005; Le Blanc et al. 2008). Furthermore, via activity on innate immune cells such as dendritic cells (DCs) and myeloid-derived suppressor cells (MDSCs) and regulatory T-cell (Aggarwal and Pittenger 2005; Yen et al. 2013), MSCs may have potential in inducing transplantation tolerance (Kim et al. 2013; Sensebe and Bourin 2009; Shi et al. 2011).

Several trials have explored the clinical utility of MSCs, both for immunosuppressive and regenerative purposes. These therapies require considerable amounts of cells. Traditionally the cells are expanded in vitro in monolayer cultures containing fetal bovine serum (FBS). The use of animal-derived components is associated with a risk of transmission of xenogenic infectious agents and immunization (Cervenakova et al. 2011; Horwitz et al. 2002; Liu et al. 2008; Sundin et al. 2007) and the use of alternative supplements or completely defined culture media would thus be preferred and is highly encouraged by the regulators (Guideline on human cell-based medicinal products, EMEA/CHMP/410869/2006 and note for guidance on minimising the risk of transmitting animal spongiform encephalopathy agents via human and veterinary medicinal products, 0EMA/410/01 rev.3). A number of studies have examined supplementing MSC cell culture media with different human blood-derived components such as platelet-derived supplements, human serum or umbilical cord blood serum (Bieback et al. 2009; Doucet et al. 2005; Fekete et al. 2012a; Schallmoser et al. 2007; Shafaei et al. 2011; Shahdadfar et al. 2005; Shetty et al. 2007). The methods employed for the production of platelet extracts are diverse (Bieback 2013). They are produced either from platelet rich plasma (PRP), which is commonly prepared by combining four buffy coat units and one AB-plasma unit with subsequent leukocyte-depletion (Schallmoser et al. 2007), or from platelet concentrates in additive solution, even expired ones (Bieback et al. 2009; Fekete et al. 2012a). Mojica-Henshaw et al. (2013) have shown that serum-converted platelet lysate (PL) can also be used as medium supplement, with the advantage that porcine-derived heparin can be omitted from the culture medium. The release of growth factors from platelets is usually induced by 2–4 freeze–thaw cycles of the platelet units or by activating the platelets with thrombin (Bieback et al. 2009). It has been suggested that at least four repeated freeze–thaw cycles might further enhance the liberation of growth factors from the platelets (Wasterlain et al. 2012). The final PL supplements are produced by either pooling several units (Schallmoser et al. 2007) or using just one unit (Horn et al. 2010).

Besides the culture medium the environment of the cells in vitro is defined by the surrounding atmosphere. Traditionally cell cultures are performed at normal atmospheric oxygen concentration (20 %) in incubators where only the carbon dioxide (CO_2_) level is regulated. The physiological oxygen concentration in human tissues is, however, much lower (2–13 %) and several stem cell types proliferate more rapidly, undergo significantly less apoptosis and DNA damage at low oxygen concentrations (Csete 2005; Estrada et al. 2012; Mohyeldin et al. 2010; Sullivan et al. 2006). Interestingly, it has been shown that low oxygen might be beneficial for the growth of MSCs at least in later passages (Dos Santos et al. 2010; Drela et al. 2014; Ren et al. 2006). In our study we wanted to test if low oxygen significantly improves cell growth also at low cell passages.

The expansion of MSCs in vitro is a necessary step to gain a sufficient number of cells for clinical needs. The culture time must, however, be kept to a minimum in order to avoid detrimental effects on the cells. Commonly used MSC cell doses that are used in the clinic for immunomodulatory purposes are in the range of 1–2 × 10^6^/kg (Ball et al. 2013; Prasad et al. 2011; Ringden et al. 2006). As even six doses may be needed per patient, it equals over 10^9^ cells for a single adult patient. It would clinically also be preferable to produce these doses from a single MSC donor. The purpose of this study was therefore to develop a reproducible culture method for clinical MSCs based on platelet-derived supplements that would yield sufficient cell numbers for at least six treatments from only 20 ml of BM.

Since many research papers have described a successful replacement of FBS by PL in MSC culture, we wanted to explore if a PL-based protocol to culture BM-MSCs could be further developed to a manufacturing method that (1) would yield high numbers (>10^9^ cells) of high quality cells after a low amount of passaging and from only 20 ml of BM and (2) could be easily and cost-effectively adapted to clinical- and GMP-grade cell manufacturing. In our current study we compared two different PL supplements, PL1 and PL2, to determine which would be better suited in a clinical cell manufacturing protocol. Our study also explored several other parameters to establish the most optimal and robust culture protocol for low passage BM-MSCs: the effect of a low oxygen concentration (3 vs 20 % concentration), the effect of repeated freeze–thaw cycles (five vs two) on the functionality of the PL and the omission of antibiotics and the sterile filtration step of the PL supplemented culture media. FBS-supplemented BM-MSC culture served as control.

We present a clinically-compliant, antibiotic-free BM-MSC culture protocol based on unfiltered PL supplement that can replace FBS also in large scale cell expansion and yielding high quantities of HLA-DR positive MSCs for clinical use.

## Materials and methods

### Platelet lysate supplements

Two different PL supplements (PL1 and PL2) were used in the study, see Table 1 for an overview of the PL supplement characteristics. All platelet units used in the study were produced by a licensed blood establishment, the Finnish Red Cross Blood Service (FRCBS) in Helsinki, Finland. The blood donors were tested according to the Finnish regulations for the preparation of blood components and in line with the regulations by the Council of Europe [Guide to the preparation, use and quality assurance of blood components, Recommendation No.R (95)15]. The blood donors tested negative for Anti-HCV, Anti-HIV-1+2, HBsAg, Syphilis, HCV-RNA, HIV-1-RNA, HBV-DNA and HAV-RNA and Parvo B19-DNA levels were below 10^5^ IU/ml. The platelet units used in this study were done following standard operating procedures within the quality system of the FRCBS and with clear release criteria involving the donor test results, platelet amounts, residual leukocytes and weight.

**Table 1:** Characteristics of the PL1 and PL2 platelet lysate supplements and their use in MSC growth media

Supplement	Composition of platelet units/bags		Platelet lysate				MSC growth media		
	Description	Platelets × 10^9a^	Lysate additive	Lysed platelets × 10^9^/ml^a^	Freeze–thaw cycles	Pool size (nr of platelet units)	Concentration of platelet lysate (%)	Lysed platelets × 10^8^/ml^a^	Concentration of plasma (%)
PL1	Platelets in plasma, 4 donors buffy coat	300	AB plasma	0.1	2	2–13	10	1.0	10
PL2	Platelets in 30 % SSP/70 % plasma, 4 donors buffy coat	300	Octaplas AB (pooled virus-inactivated fresh frozen plasma)	15	5	15	0.5 (+ 2.5 % Octaplas AB)	0.8	3

^a^Mean numbers

PL1 was essentially produced as described by Schallmoser et al. (2007). PRP units were produced by combining buffy coats of four individual blood donors with one unit of AB-plasma. The platelets were separated by centrifugation and the remaining leukocytes were removed by filtration, after which the units were frozen at −20 °C. PL1 pools were subsequently produced by pooling 2–13 thawed PRP units and the combined pool was then frozen in aliquots at −20 °C. Thus each PL1 pool originated from buffy coats of 8–52 individual blood donors and was frozen twice during production. The efficiency of each PL1 pool to support MSC growth was tested in a 5–7 day proliferation assay and with 2–4 different BM-MSC batches as responder cells (Table 2). The pools were released for use if the responder cells exhibited a population doubling of 3 and above during the 5–7 day assay. To produce PL2, expired bags of platelet concentrates in 30 % additive solution SSP (MacoPharma, Langen, Germany and 70 % plasma) were centrifuged at 3,200×*g* for 20 min at room temperature. The pellets were suspended in 20 ml of pooled frozen AB-plasma (Octaplas AB, Octapharma AG, Lachen, Switzerland) per bag of platelets, frozen at −70 °C and subsequently thawed in a +37 °C water bath. After five freeze–thaw cycles the platelets were centrifuged at 3,200×*g* for 20 min at room temperature and the supernatants were collected and stored at −20 °C. Each PL2 lysate was tested for efficiency by supporting MSC growth at least at the same levels as FBS before producing the PL2 pool. The PL2 pool for this study was prepared by pooling 15 individual PL2 units thus originating from 60 individual donors.

**Table 2:** Functionality testing of the platelet lysate 1 (PL1) supplement pools based on MSC population doubling (PD) in a 5–7 day proliferation test. MSCs from 2 to 4 different donors served as responder cells

PL1 pool	Number of PRP units	Number of donors	Number of PDs (mean)	Range of PDs
Pool 1	2	8	4.44	3.0–5.88
Pool 2	3	12	4.54	3.17–5.91
Pool 3	6	24	4.82	3.46–6.19
Pool 4	4	16	5.27	4.75–5.78
Pool 5	4	16	5.00	4.32–5.83
Pool 6	11	44	5.11	4.39–5.81
Pool 7	13	52	3.82	3.32–4.32

All pools of PL were also tested for sterility by BacT/ALERT (bioMérieux, SA, Marcy-I’Etoile, France). When thawed for use the supplements were finally centrifuged at 3,200×*g* for 20 min at room temperature (RT) immediately before use and the supernatant was used.

### Bone marrow harvest

BM was collected from 15 voluntary healthy donors, aged 20–40, after written informed consent. The study was approved by the Ethical Committee of the Hospital District of Helsinki and Uusimaa. 20 ml of BM was drawn under local anaesthesia from the posterior iliac crest into heparinized syringes. The samples were processed within 2 h from harvest. For mononuclear cell (MNC) isolation the BM samples were diluted 1:3 with DPBS CTS™ (Life Technologies, Thermo Fisher Scientific, Waltham, MA, USA) and 2 mM EDTA (pH 7.2) or later on in the study with Versene (EDTA) 0.02 % (Lonza, Basel, Switzerland) and layered on Ficoll-Paque PREMIUM (GE Healthcare Bio-Sciences, Uppsala, Sweden) and centrifuged at 400×*g*, 40 min at RT. The BM-MNCs were collected, washed with DPBS CTS™-EDTA/Versene and counted as described in the next chapter.

### Culture of MSCs

The BM-MSC basal medium consisted of D-MEM (low glucose, Life Technologies), 100 U/ml penicillin, 100 µg/ml streptomycin (Life Technologies) and 40 IU/ml heparin (Heparin LEO^®^ 5000 IE/KY/ml, Leo Pharma, Malmö, Sweden) to prevent gelatinization and avoid clots. Later on in the study, the antibiotics were omitted when process developmental work was transferred to a cleanroom environment. The basal medium was supplemented either with 10 % PL1 or with 0.5 % PL2 and 2.5 % AB-plasma (Octaplas, Octapharma), see Table 1. The control medium consisted of basal medium and 10 % FBS without heparin. The PL1 and PL2 containing media were initially sterile filtrated with a 0.22 µm filter before use in culture. Later on in the study and opposite to other published protocols (Schallmoser et al. 2007), we omitted the filtration process with the PL1 supplemented medium as clinically-compliant and xenofree filters were not available in the culture scale needed. The BM-MNCs were plated in culture vessels at 400,000 cells/cm^2^ and were placed in a humidified incubator at +37 °C, 5 % CO_2_ with either 3 or 20 % oxygen. After 3 days the wells were washed four times with DPBS CTS™ to remove non-adherent cells and the medium was changed. The medium was changed twice weekly until the cells reached 90 % confluency. At each passage the vessels were washed with DPBS CTS™ and the cells were detached with TrypLE™ Express (Life Technologies) and later on in the study with TrypLE™ Select CTS™ (Life Technologies) and reseeded at 1,000 cells/cm^2^. The cell number and viability of the cells was determined using a Bürker-chamber or NucleoCounter NC-100™ (ChemoMetec, Allerod, Denmark). Aliquots of cells in interim phases of culture (p0, p1) were frozen in liquid nitrogen and thawed and cultured for analysis if needed. Freezing of the cells was done in the initial phase of the study in 50 % of the appropriate proliferation medium, 40 % FBS and 10 % DMSO Hybri-Max™ (Sigma-Aldrich, Ayrshire UK), but later on in the study in 90 % human albumin (Albunorm 200 g/l, Octapharma) and 10 % Cryoserv^®^ DMSO (Bioniche Pharma, Lake Forest, IL, USA). All proliferation kinetic and long-term culture studies were done with cells without interim freezing.

The PL1-supplemented culture protocol at 20 % oxygen concentration was chosen for further process development work and was developed towards a clinically and GMP-compliant method. Large-scale MSC culture was developed using 1-, 2- and 5-STACK culture vessels (CellStacks^®^, Corning Inc., Corning, NY, USA). During the large-scale process development phase, all the materials were clinically- and GMP-compliant. In the final process development stage of the study, also the filtering of the medium and the antibiotics were omitted from the culture medium as the cell production was performed in class A cleanroom environment.

### Proliferation kinetics

To determine the colony forming unit-fibroblasts (CFU-F) content of the starting BM-MNC material, the BM-MNCs were plated in six-well plates (Corning Inc.) at 400,000 cells/cm^2^ and cultured for 5–10 days at +37 °C, 5 % CO_2_, 20 % oxygen. The cells were then fixed with methanol and stained with Giemsa solution (Merck KGaA, Darmstadt, Germany).

The number of population doublings (PD) was calculated using the formula N_H_ = 2^PD^ × N_1_ in which N_H_ is the number of harvested cells/cm^2^ and N_1_ is the number of seeded cells/cm^2^. PD is then determined as PD = log_2_ (N_H_/N_1_). At passage zero the CFU-F number in the original BM aspirate was the initial N_1_. The PD time was calculated as length of passage (days)/number of PDs reached during passage.

### Genetic stability

Cells at passage two were plated in cell culture flasks (Corning) at 1,000 cells/cm^2^ and grown to 70–80 % confluency for karyotyping. At least 20 mitotic cells were analysed from each sample by conventional G-band analysis by an accredited laboratory (Medix Laboratories, Espoo, Finland). Results were informed as either normal karyotype (46, XX or 46, XY) or abnormal (with corresponding chromosomal abnormality).

### Differentiation assays

To assess the adipogenic and osteogenic potential of the BM-MSCs, cells from passage two were seeded onto 12-well plates (Nunc) at 3,000 cells/cm^2^ and the cells were grown to confluency. For adipogenic differentiation the cells were changed into an adipogenic induction medium for 2–3 days after which the cells were incubated in terminal adipogenic medium for 1–2 weeks. The induction medium and terminal differentiation medium consisted of the same adipogenic basal medium containing alpha-MEM Glutamax, 10 % FBS, 20 mM HEPES, 100 U/ml penicillin, 100 µg/ml streptomycin (all from Life Technologies), 0.5 µg/ml insulin (Promocell, Heidelberg, Germany) and 0.1 mM indomethacin (Sigma-Aldrich, St Louis, MO, USA). In addition the induction medium contained 0.2 mM 3-isobutyl-1-methylxanthine (IBMX), and 0.4 µg/ml dexamethasone (both from PromoCell) and the terminal differentiation medium contained 3 µg/ml Ciglitazone (PromoCell) (Suila et al. 2011). After differentiation the cells were fixed with 4 % paraformaldehyde (PFA) for Sudan III (Sigma-Aldrich) staining.

For osteogenic differentiation the cells were cultured in osteogenic medium for 3–4 weeks. The osteogenic medium consisted of α-MEM supplemented with 10 % FBS, 20 mM HEPES, 2 mM l-glutamine (all from Life Technologies), 0.1 µM dexamethasone, 10 mM β-glycerophosphate, 0.05 mM l-ascorbic acid-2-phosphate (all from Sigma-Aldrich) and penicillin–streptomycin (Life Technologies). Animal serum-free osteogenic differentiation medium consisted of D-MEM Glutamax (low glucose, Life Technologies), 10 % PL1 and 40 IU/ml heparin (LeoPharma), 0.1 µM dexamethasone, 10 mM β-glycerophosphate, 0.05 mM l-ascorbic acid-2-phosphate. After differentiation the cells were fixed with 4 % PFA for von Kossa staining.

For chondrogenic differentiation 200,000 cells from passage two were pelleted into a micromass by centrifugation at 400×*g* for 5 min in 15 ml conical polypropylene tubes. The pellets were cultured for 2 weeks in chondrogenic medium that consisted of D-MEM (high glucose, containing 0.1 mM pyruvate, Life Technologies), supplemented with 10 ng/ml transforming growth factor beta (TGF-β), 0.1 µM dexamethasone, 0.1 mM l-ascorbic acid-2-phosphate, 40 µg/ml l-proline (all four from Sigma-Aldrich), 1 × ITS + premix (BD Biosciences, Bedford, MA, USA) and penicillin–streptomycin (Life Technologies). The cell pellets were fixed with 10 % formalin, embedded in paraffin, cut into sections and stained with Alcian blue (Sigma-Aldrich) and Nuclear fast red (Merck).

### Flow cytometry analysis

For analysis of immunophenotype the cells were detached with TrypLE™-express (Life Technologies) and washed with FACS buffer solution (0.3 % BSA (Sigma-Aldrich) in PBS-2 mM EDTA). Fluorescein isothiocyanate (FITC), phycoerythrin (PE) or allophycocyanin (APC)-conjugated antibodies against CD13, CD14, CD19, CD29, CD44, CD45, CD49e, CD73, HLA-DR, HLA-ABC (all from BD Pharmingen, San Diego, CA, USA), CD34 (Miltenyi Biotec GmbH, Gladbach, Germany), CD90 (StemCell Technologies Inc., Vancouver, BC, Canada) and CD105 (Abcam, Cambridge, UK) were used for direct labelling of the cells. Appropriate FITC-, PE- and APC-conjugated isotype controls (all from BD Biosciences) were used. *N*-Glycolylneuraminic acid (Neu5Gc, Gc-Free, Biolegend, San Diego, CA, USA) staining was performed according to manufacturer’s instructions followed by AlexaFluor 488 labelled goat anti-chicken secondary antibodies (Molecular Probes, Invitrogen, Eugene, OR, USA). 1 µM Sytox Blue (Molecular Probes) was used to exclude dead cells. The cells were analysed with FACSAria flow cytometer and FACSDiva 5.0.3 (BD, San Jose, CA, USA) and FlowJo 7.6.1 softwares (TreeStar, Ashland, OR, USA).

### Immunosuppression assay

To interrogate the capacity of MSCs to suppress T-cell proliferation the cells were co-cultured with peripheral blood mononuclear cells (PB-MNC). 1.5 × 10^5^, 0.75 × 10^5^ or 0.3 × 10^5^ MSCs were suspended in RPMI 1640 medium supplemented with 5 % FBS and penicillin–streptomycin (all from Life Technologies) and plated in 48-well plates. The MSCs were allowed to adhere onto the plates in an incubator before the PB-MNCs were added.

PB-MNCs were isolated from buffy coats by Ficoll-Paque Plus (GE Healthcare, Helsinki, Finland) gradient centrifugation and labelled with 2.5 µM CFSE [5(6)-carboxyfluorescein diacetate *N*-succinimidyl ester, Molecular Probes] in 0.1 % HSA-PBS (human serum albumin, Sanquin, Espoo, Finland) for 5 min at room temperature. 1.5 × 10^6^ labelled PB-MNCs were then added to the co-culture. For T-cell activation 0.1 µg/ml of CD3 antibody (clone Hit3a, BioLegend, San Diego, CA, USA) was added to the wells. T-cell proliferation was recorded after 4 days of co-culture as dilution of fluorescent dye by flow cytometry. The division index (Flow Jo software v.7.6.1) was used to represent the extent of cell division.

### Statistical analysis

All data are presented as the mean ± standard deviation (SD) unless mentioned otherwise. The differences in mean values were tested by one-way analysis of variance (ANOVA) and the Tukey’s post hoc test. The data were analyzed with GraphPad Prism software version 5.04 (GraphPad Software, La Jolla, CA, USA) and statistical programming software R (version 2.14.0). The differences were considered significant when *p* < 0.05.

## Results

### BM processing

The BM samples were initially aspirated into heparinized syringes, but since some of the BM samples contained clots the standard operating procedures were modified to also include 2,500 IU of heparin per 10 ml syringe. The MNC yield after gradient centrifugation was 2.98 × 10^6^ ± 1.310^6^/ml of BM.

### Small pools of PL1 are as efficient as larger pools

Each pool of PL1 was tested using 2–4 individual MSC batches as responder cells. Tested pools consistently supported the expansion of MSCs through 3.0–6.2 PDs in a 5–7 day proliferation assay (Table 2). Pools produced from two PRP units were as efficient as ones from 13 units. For practical reasons, large pools (e.g. pools of 8–10 PRP units) are preferable.

### PL1 provides a good support for MSC growth

**Fig. 1:**
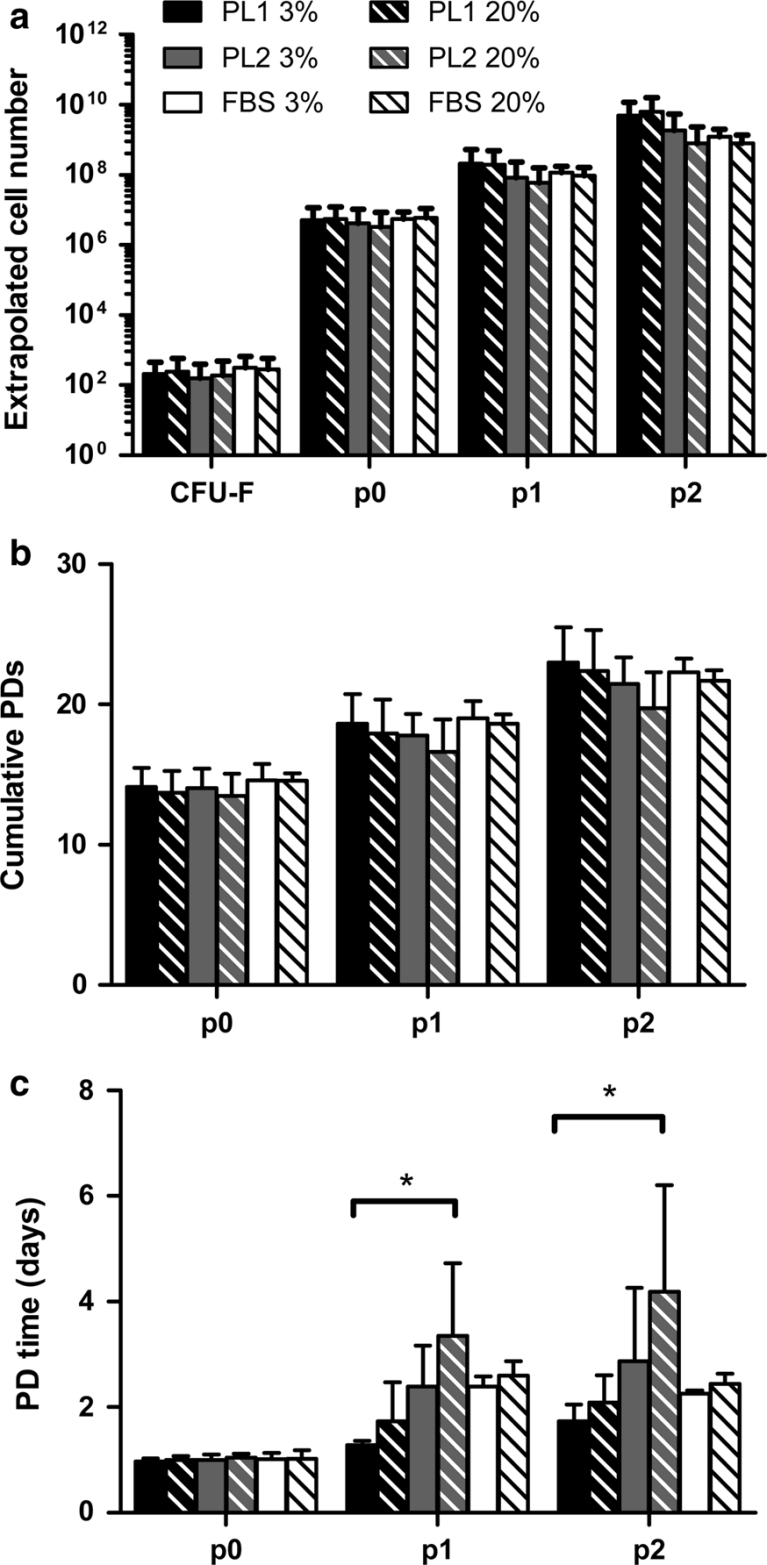
Proliferation kinetics of cells cultured in six different culture conditions. **a** Extrapolated cell yield from 20 ml of BM up to passage two. **b** Cumulative PDs and **c** PD time at different passages from BM-MNC to passage 2. **b**, **c** The CFU-F numbers were used as the starting number of the cells at passage 0. Data are represented as mean ± SD (n = 4), except for FBS cultures where only mean values are shown (n = 2)

We compared the ability of three different culture medium supplements, PL1, PL2 and FBS, to support MSC growth up to passage two in 20 and 3 % oxygen. There was no statistical difference in total cell yield or cumulative PD between different culture conditions (*p* = 0.42 and 0.99, respectively, Fig. 1a, b). However, cells cultured in PL1-medium reached the highest cell yields (extrapolated cell numbers from 20 ml of BM were 6.31 × 10^9^ ± 9.82 × 10^9^ in 20 % oxygen and 4.81 × 10^9^ ± 6.78 × 10^9^ in 3 % oxygen) and the highest PDs (22.4 ± 2.9 PDs in 20 % oxygen and 23.0 ± 2.5 PDs in 3 % oxygen) within the shortest PD times (2.1 ± 0.5 days in 20 % oxygen and 1.7 ± 0.3 days in 3 % oxygen at passage 2), see Fig. 1. When PL1 cultured cells were compared with PL2 the PD time was significantly shorter regardless of oxygen conditions (*p* = 0.015 by one-way ANOVA, Fig. 1c). There was no statistical difference in the PD time between PL1 and FBS cultured cells. The use of 3 % oxygen led to a trend of shorter PD times with each medium reaching statistical significance only between cells cultured in PL1-medium in 3 % oxygen versus PL2-medium in 20 % oxygen (*p* = 0.04, Fig. 1c).

**Fig. 2:**
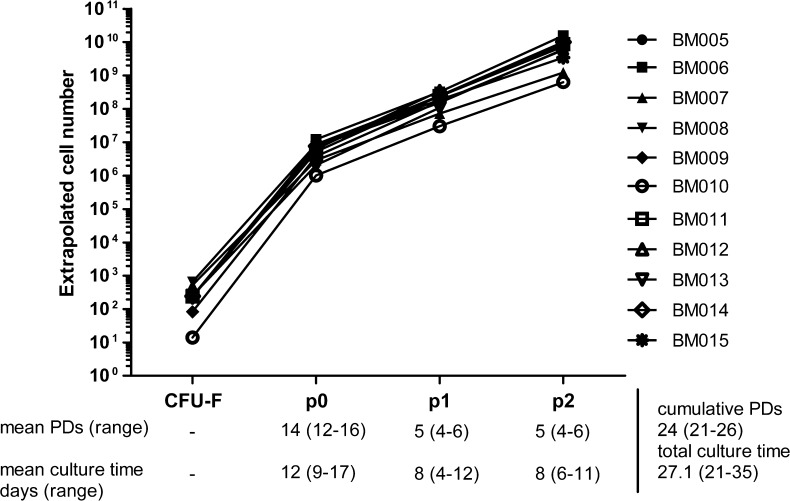
Extrapolated MSC yield of 11 BM-MSC batches cultured in PL1-medium at normal atmospheric oxygen conditions. The mean PDs and culture time are shown under each passage and the cumulative mean PD number and the mean value of total culture time are shown on the *right-hand side* of the table. The range is shown in parenthesis

**Fig. 3:**
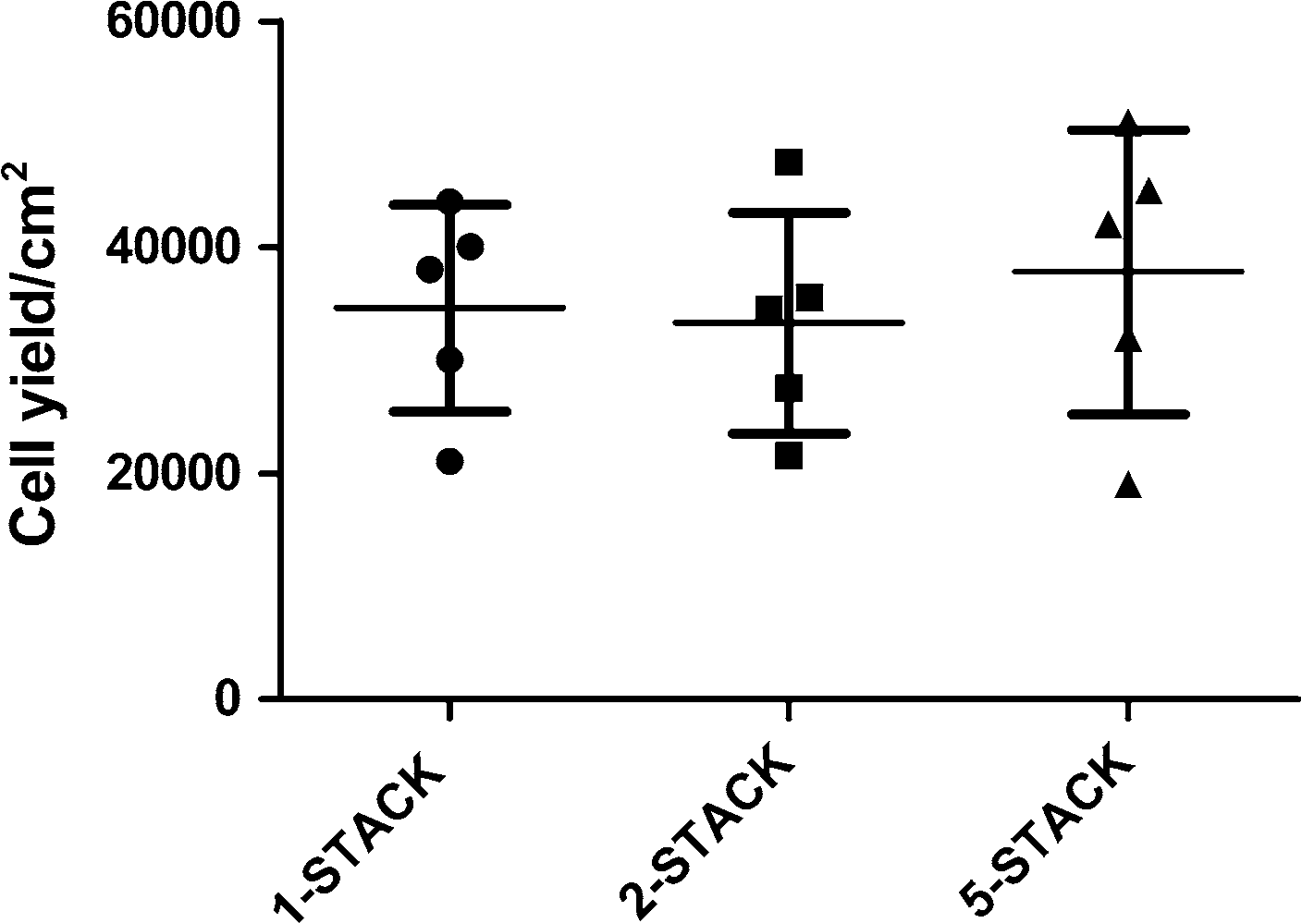
Cell yields of PL1-cultured BM-MSCs in large-scale vessels (1-, 2- and 5-STACK) in passage 2. Cells were seeded 1,000 cells/cm^2^ and cultured for one passage. Data show the cell yield/cm^2^ ± SD (*p* = 0.79, n = 5)

After these initial experiments, we chose to test the suitability of the PL1-medium and 20 % oxygen for large-scale expansion using suitable large cell culture vessels of MSCs with 11 subsequent bone marrow samples. The mean CFU-F number/ml of BM was 17.30 ± 10.83, representing 0.0001–0.0009 % of BM-MNCs. We set 10^9^ cells at passage 2 as the goal for the cell culture process, thus sufficient for 6 cell doses for a patient of 80 kg. As can be seen in Fig. 2, the goal of 10^9^ cells was reached at passage two in 73 % of the BM-MSC batches (8/11) and within 21–26 PDs (mean cell number 6.6 × 10^9^ ± 4.74 × 10^9^). Clotted BM aspirates performed poorly and was identified as the primary reason behind a lower cell yield as passage 2. Passage two was reached within 21–35 days (Fig. 2). All tested large cell culture vessels performed equally well and with consistent cell yield/cm^2^ (*p* = 0.79) indicating a robust and even cell expansion in the chosen large cell culture vessels and independent of the number of layers in the vessel (Fig. 3). The karyotype of passage two cells was normal (46, XX or 46, XY) in all 11 BM-MSC batches.

**Fig. 4:**
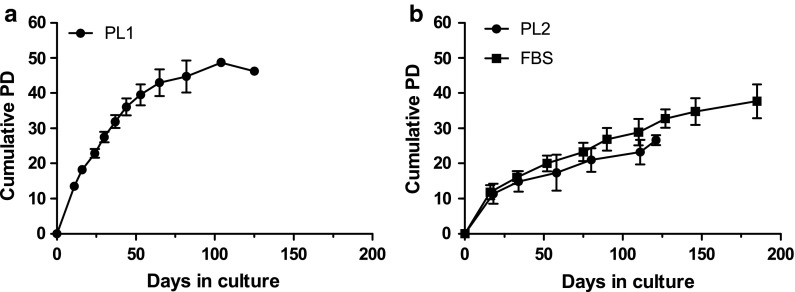
Growth kinetics of MSCs in long term culture until growth arrest. Cells were cultured **a** in PL1- (n = 3), **b** in PL2- and in FBS- (same donors for both, n = 16) supplemented media from primary cultures to senescence. Data show mean values ± SD

Long-term cultures revealed that the proliferation of cells cultured in PL1-medium was arrested after 46 PDs and was superior to the cells cultured in PL2-medium and FBS-medium, which ended proliferation after 27 PDs and 38 PDs, respectively (Fig. 4). Total culturing time for cells in PL1-medium was 125 days until growth arrested, whereas cultures in PL2 and FBS took 121 and 185 days, respectively (Fig. 4).

### MSC characterization

**Fig. 5:**
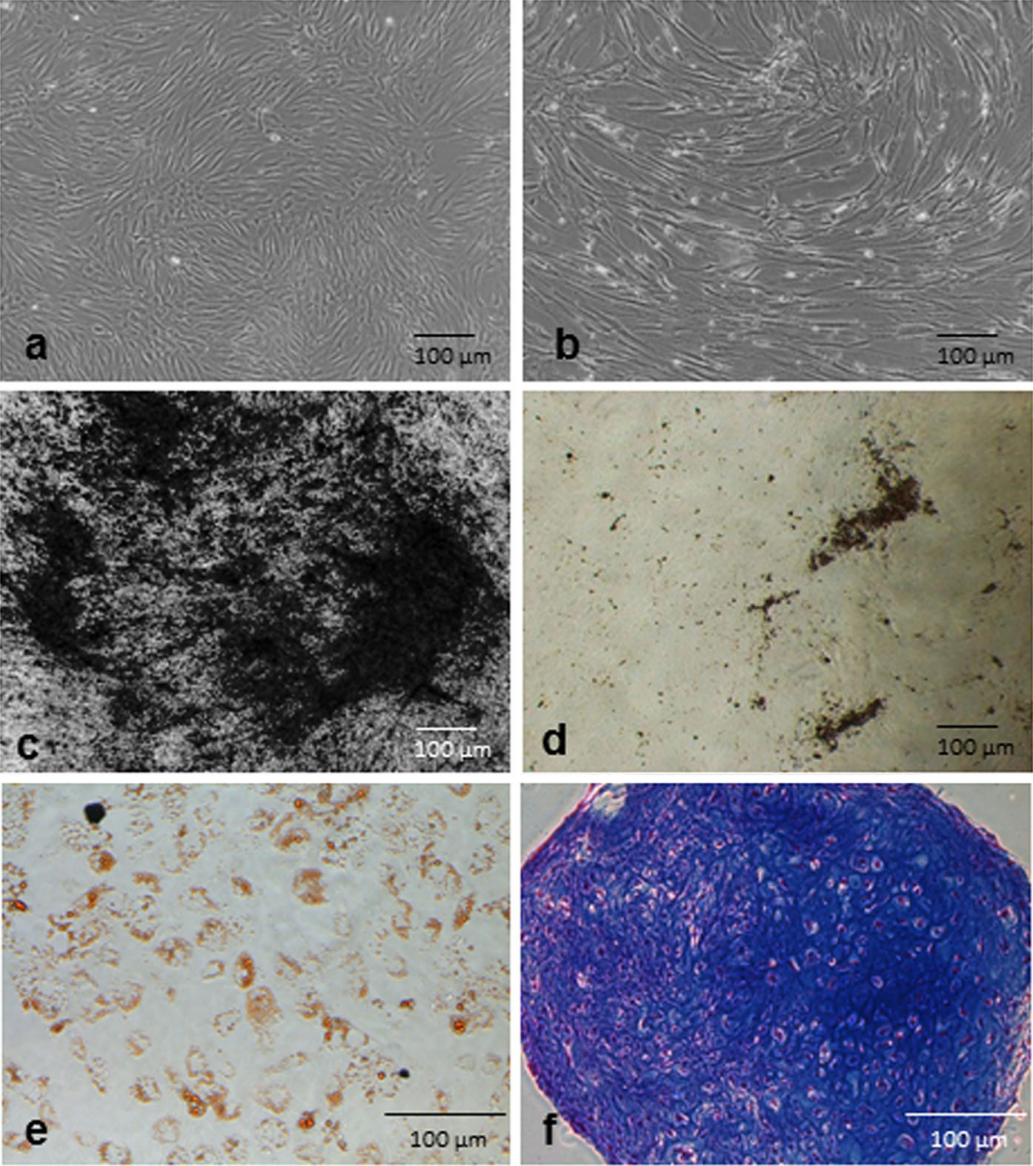
Representative photographs of cell morphology and differentiation. Morphology of passage 2 cells was normal spindle shaped for both **a** PL1- and **b** PL2-medium cultured BM-MSCs, but the PL2-cultured cells appeared to have a slightly larger morphology. Von Kossa staining of osteogenic differentiated BM-MSCs in **c** PL1-supplemented differentiation medium and in **d** FBS-supplemented differentiation medium. **e** Sudan III staining indicated the adipogenic differentiation potential of the samples and **f** Alcian blue staining indicated the chondrogenic potential of the cells grown in PL1-medium

The morphology of the cells was typical for MSCs with slight size difference between PL1- and PL2-medium cultured cells with PL1 cells appearing smaller (Fig. 5a, b).

All BM-MSC batches tested displayed typical MSC differentiation capacity along the adipogenic, osteogenic and chondrogenic lineages at passage two (Fig. 5c–f). Von Kossa staining of osteogenic cultures revealed a more intense calcium deposition in PL1 containing differentiation medium compared to those differentiated in FBS containing differentiation medium (Fig. 5c, d).

**Fig. 6:**
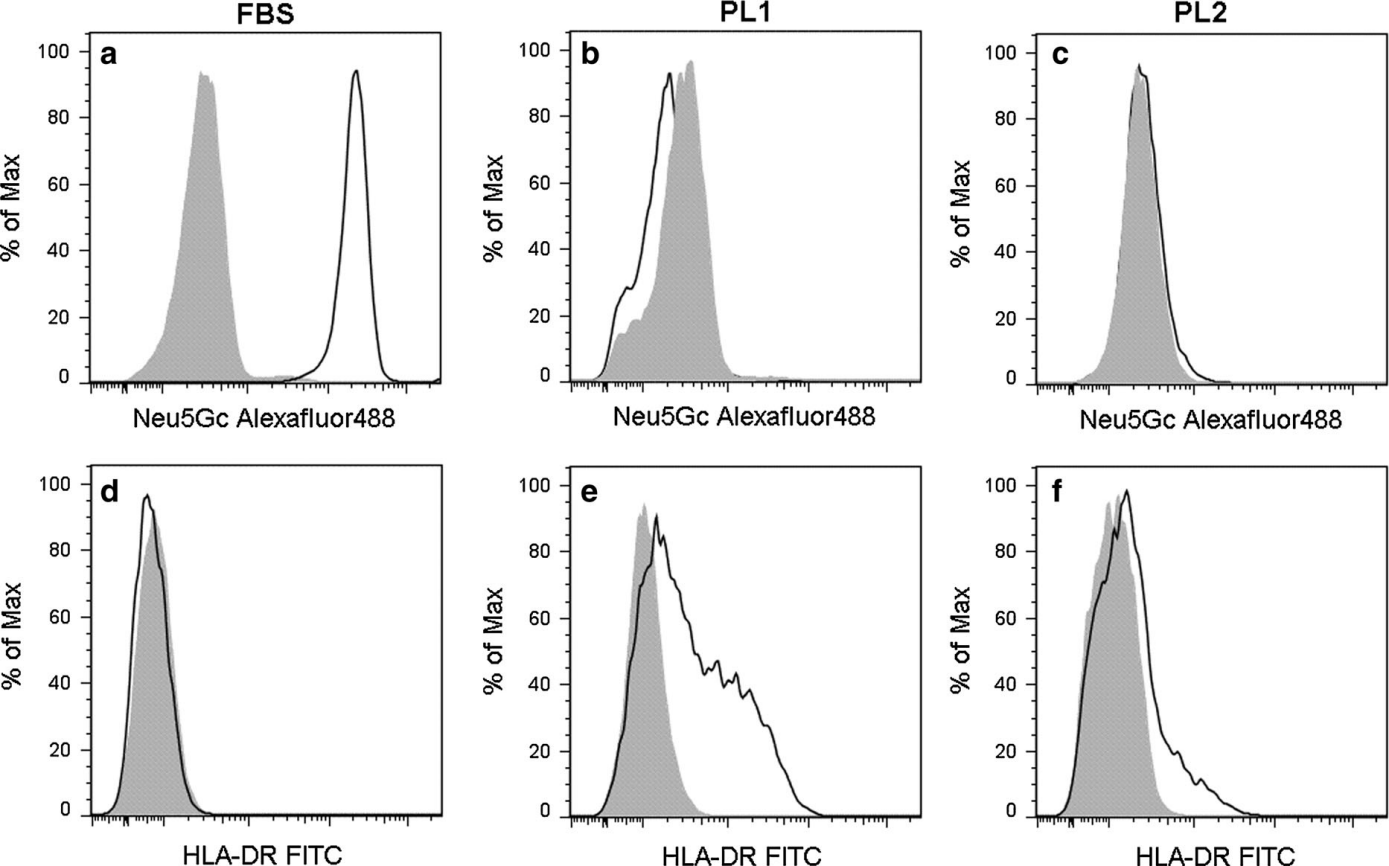
Xenoantigenic Neu5Gc contamination and expression of HLA-DR on MSCs. BM-MSCs cultured in **a** FBS-containing medium stained positively with anti-Neu5Gc whereas cells cultured in **b** PL1- and in **c** PL2-media did not have Neu5Gc on their surface. HLA-DR was not expressed on BM-MSCs cultured in **d** FBS-containing medium but was found on cells cultured in **e** PL1- and **f** PL2-medium. Filled histograms represent the unspecific/isotype controls and the solid line the specific staining

The immunophenotype of the cells from all culture conditions was typical for MSC (Dominici et al. 2006) with the exception of the expression of HLA-DR (Fig. 6). The cells were negative for hematopoietic markers and they expressed typical MSC markers on their surface [CD13, CD29, CD44, CD49e, CD73, CD90, CD105 and HLA-ABC (Table 3)]. The BM-MSCs cultured in PL1 supplemented culture media were consistently HLA-DR positive (7.5–66.1 %, Table 3). FBS cultured BM-MSCs were HLA-DR negative (Fig. 6). The xenoantigen Neu5Gc was detected on the cell surface of cells cultured in the presence of FBS but not on cells cultured in PL1- and PL2-medium (Fig. 6).

**Table 3:** Immunophenotype of BM-MSCs cultured in PL1-medium

Surface antigen	Mean %	Max %	Min %	n
CD44	98.9	100.0	95.6	11
CD49e	99.0	100.0	95.5	11
CD13	99.0	100.0	95.6	11
CD90	99.9	100.0	99.3	11
CD73	99.9	100.0	99.2	11
CD29	99.7	100.0	98.5	11
CD105	99.0	100.0	96.5	11
HLA-ABC	99.6	100.0	98.6	11
CD14	<1	<1	<1	5
CD19	<1	<1	<1	5
CD34	<1	1.8	<1	5
CD45	<1	<1	<1	5
HLA-DR	26.8	66.1	7.5	11

Mean percentage and maximum and minimum values of positive cells for each antigen are shown

### Differently cultured MSCs have similar capacity to suppress T-cell proliferation

**Fig. 7:**
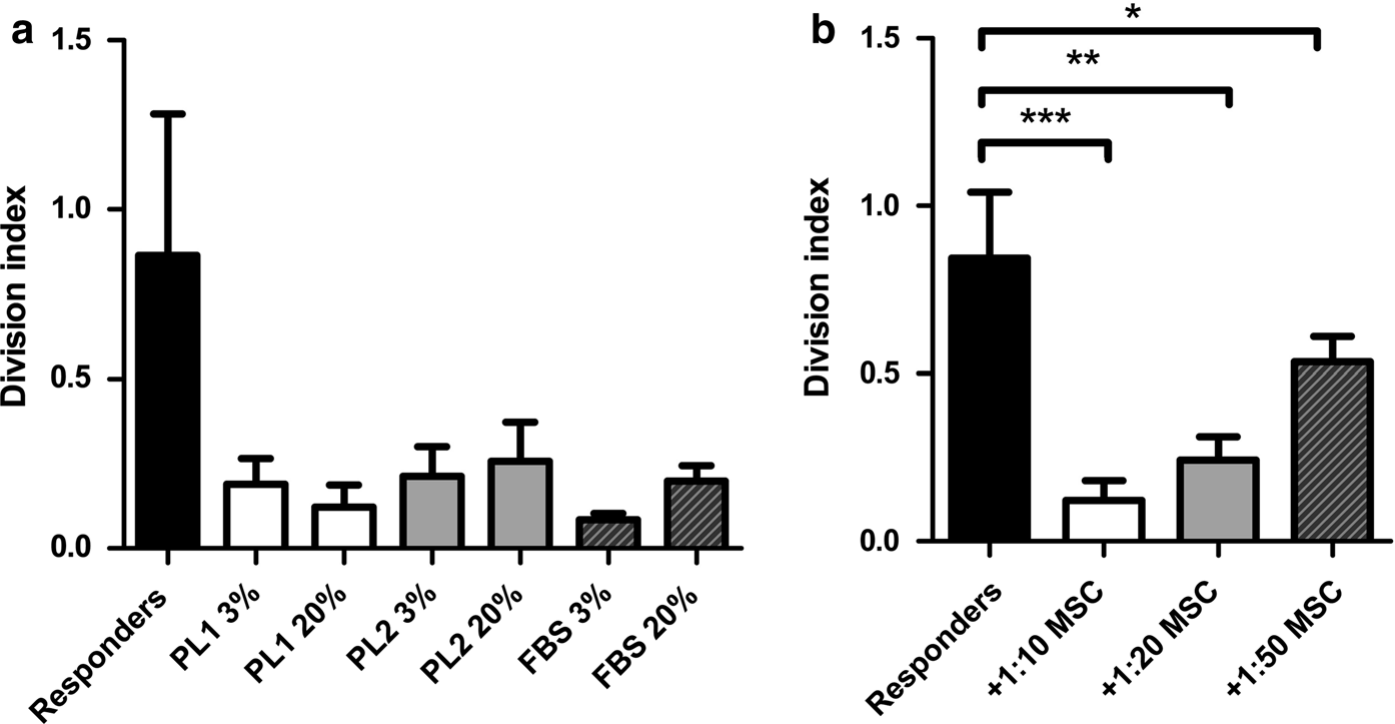
Immunosuppressive capacity of BM-MSCs cultured in different culture conditions and the dose dependent capacity of the cells to suppress T-cell proliferation. **a** The results of immunosuppressive capacity of the cells cultured in six different culture conditions at a ratio of MSC:MNC 1:10. Differences between cells from different culturing conditions were statistically non-significant (0.14 by one-way ANOVA). **b** The suppressive capacity of MSCs cultured in PL1-medium was dose dependent. Division index of three independent experiments (mean + SD) is shown indicating the average number of cell divisions. Statistical significance is tested using one-way ANOVA and Tukey’s post hoc test *p < 0.05, **p < 0.01, ***p < 0.001

The capacity of MSCs to suppress T-cell proliferation was tested in co-culture with PB-MNCs that were stimulated with an anti-CD3 antibody. The MSCs cultured in different conditions were all able to suppress T-cell proliferation at a 1:10–1:50 suppressor:effector ratio. Dose dependence of the suppression was demonstrated with MSCs cultured with PL1 (Fig. 7).

## Discussion

Various animal serum-free culture methods utilizing platelet extracts to support MSC expansion have been published (Bernardo et al. 2007; Bieback et al. 2009; Capelli et al. 2007; Doucet et al. 2005; Mojica-Henshaw et al. 2013; Schallmoser et al. 2007). We wanted to explore if a PL-based protocol to culture BM-MSCs could be further developed to a manufacturing method that (1) would yield high numbers (>10^9^ cells) of high quality cells after a low amount of passaging and from only 20 ml of BM and (2) could be easily and cost-effectively adapted to clinical- and GMP-grade cell manufacturing.

We compared two different platelet-derived supplements, platelet lysate PL1 versus PL2, produced by two different methods and lysed with different amounts of freeze–thaw cycles. Our study also explored the effect of a low oxygen concentration on BM-MSCs. FBS-supplemented BM-MSC culture served as control. Since PL2 was more concentrated (Table 1), we used 10 % of PL1 and 0.5 % of PL2 in the basal medium to receive a comparable concentration of lysed platelets in the final medium, 1 × 10^8^ platelets/ml in PL1 and 0.8 × 10^8^ platelets/ml in PL2 containing medium, which is in accordance with other studies (Lange et al. 2007; Muller et al. 2006). Lange et al. (2007) showed that the proliferation is reduced if the platelet concentration of the starting material is less than 0.8 × 10^8^ platelets/ml in the final medium. The PL1-medium had the best capacity for promoting MSC proliferation. This is in agreement with reports comparing PL supplemented media with FBS and thrombin-activated platelet release (Ben Azouna et al. 2012; Bernardo et al. 2007; Bieback et al. 2009; Capelli et al. 2007; Carrancio et al. 2008; Doucet et al. 2005; Griffiths et al. 2013; Horn et al. 2010; Lange et al. 2007; Salvade et al. 2010; Schallmoser et al. 2007). The PL1-supplemented BM-MSC cultures consistently reached clinically relevant numbers of cells within two passages. If BM aspirates contained clots, however, the cultures were less successful, emphasizing the importance of heparin during the BM harvest. 0.5 % PL2 as medium supplement was less efficient than 10 % FBS supplemented medium, which is in agreement with Bernardo et al. (2007).

The main differences between the two PL supplements tested were (1) the higher plasma concentration in PL1 and that (2) PL2 was subjected to several freeze–thaw cycles (5 vs 2, Table 1). Our results imply that a higher plasma concentration in the MSC culture media might be beneficial. The importance of the plasma fraction for the initial outgrowth of MSC colonies has also been demonstrated by Horn et al. Horn et al. (2010) who showed that PL alone could not support CFU-F formation. We could, however, not see any benefits of a high number (5) of freeze–thaw cycles. Repeated freezing and thawing might actually negatively affect the growth factor content of platelet-derived supplements (Mojica-Henshaw et al. 2013), and the high amount of freeze–thaw cycles during the manufacture of PL2 may have inactivated some critical components of the supplement used in our study. Although the cell yields did not significantly differ between the PL1 and PL2 supplemented protocols, PL1 was superior in that PD time was shorter (*p* = 0.015). It is also worth considering that residues from the virus-inactivated pooled plasma in the PL2-supplemented protocol might affect the proliferation of the BM-MSCs.

Although autologous PL may be preferable in specific situations to minimize immunologic side-effects and viral infections, large pools are preferable for large scale expansion due to their consistent performance and easier logistics. Individual human PLs (hPLs) differ in their cytokine profile as well as their ability to support MSC proliferation (Horn et al. 2010). Mojica-Henshaw et al. (2013) showed that different PL lots produced from at least 5–6 PRP units do not differ much in growth factor content and this variability is expected to decline with even larger pools. In our study the PL1 pools were produced from 2 to 13 platelet units, i.e. platelets from 8 to 52 individuals, and all pools performed consistently in supporting MSC expansion (more than 3 PDs in a 5–7 day assay). We could also conclude in the final stages of the study that filtering of the PL-supplemented media is not needed if the platelet units and pools are produced with high quality standards and according to strict GMP.

Divergent results have been published about the influence of different culture supplements on the number of CFU-Fs in the primary cultures. Some studies have shown that the different culture supplements do not influence the number of proliferating multipotent stem cells, but rather their expansion efficiency. (Ben Azouna et al. 2012; Doucet et al. 2005; Horn et al. 2010; Schallmoser et al. 2007). Our results are in accordance with these data. Some have reported that PL containing media might also increase the number of CFU-Fs (Lange et al. 2007; Salvade et al. 2010). There are also conflicting results regarding the effect of oxygen concentration on the CFU-F number (Fehrer et al. 2007; Lennon et al. 2001). Oxygen concentration is often an ignored component of the culture conditions and cells are kept in normal atmospheric oxygen, the only controlled gas being CO_2_. The atmospheric 20 % oxygen concentration is considerably higher than the 2–9 % oxygen concentration in the natural niche of MSCs (Haque et al. 2013; Mohyeldin et al. 2010). It has been shown in some reports that low oxygen shortens the expansion time of MSCs (Carrancio et al. 2008; Estrada et al. 2012; Grayson et al. 2007) and it has been claimed that the MSC yield could be maximized in low oxygen and the culture time reduced when expanding MSCs at clinical scale (Dos Santos et al. 2010). In these studies the beneficial effect of low oxygen is shown with cells that are from passage 2 or more. Albeit we saw a trend of shorter PD time at p2 at 3 % oxygen, the effect of low oxygen on BM-MSC proliferation was not significant. Others have also found that oxygen concentration does not influence the proliferation of low passage cells (Fehrer et al. 2007; Karlsen et al. 2011), but PD time is clearly shortened at later passages (Tsai et al. 2011). Low oxygen may have other benefits however, as it may reduce oxidative stress and genetic instability (Chen et al. 1995; Estrada et al. 2012). However, the practicality and economic concerns of the culture protocol are decisive factors in large scale MSC manufacturing. The possible advantage of culturing the cells in low oxygen concentration may be neutralized by its added work and cost. Our results suggest that as long as the cells are expanded only for a few passages the harmful effects of 20 % oxygen are minimal.

The PL1-supplemented culture protocol at 20 % oxygen concentration was chosen for further process development work and was developed towards a clinically and GMP-compliant method. We found that MSCs can be cultured with this protocol at large scale using CellStacks^®^ and the method consistently yields MSCs of uniform quality. The cells also essentially fulfilled the MSC minimal criteria set by Mesenchymal and Tissue Stem Cell Committee of International Society of Cell Therapy (ISCT) (Dominici et al. 2006) with one exception, the HLA-DR expression. The cell surface expression of the class II HLA molecule HLA-DR was consistently positive after culturing in PL, but was absent in cells cultured in FBS (Fig. 6). An induction of HLA-DR expression has been previously reported on MSCs cultured in FBS after cytokine stimulation (Bocelli-Tyndall et al. 2010; Le Blanc et al. 2007; Romieu-Mourez et al. 2007; Turnovcova et al. 2009), and some recent reports have suggested a low expression of HLA-DR on MSCs cultured in PL (Fekete et al. 2012b; Tarte et al. 2010). The expression of HLA-DR has, however, been omitted from numerous papers describing MSC culture in PL (e.g. Doucet et al. 2005; Horn et al. 2010) and we speculate that the HLA-DR expression data have been omitted since it does not fulfill the ISCT minimal criteria for MSCs. The ISCT criteria are formulated using cells cultured in presence of FBS and may not reflect MSCs cultured in differently supplemented media. We found, as also reported by others, that MSCs expressing HLA-DR molecules also possess immunosuppressive capacity, possibly because they lack expression of co-stimulatory molecules (CD80 and CD86) and thus do not elicit an immune reaction (Le Blanc et al. 2007; Menard et al. 2013; Sotiropoulou et al. 2006; Tarte et al. 2010; Tse et al. 2003). Tarte et al. (2010) reported that MSCs that express HLA-DR are poorly immunogenic and efficiently suppress T-cell proliferation in mixed lymphocyte reaction (MLR) and the expression of HLA-DR should not be considered a critical release criterion for MSCs. Duijvestein et al. (2011) hypothesized that pre-activation of MSCs with INF-γ that also induces the expression of HLA-DR could lead to more rapid clinical response and hence a lower dose of cells is needed. MSCs may thus receive beneficial activation signals from PL. The functional consequences of the cell surface expression of HLA-DR is still unknown and would need further investigations, but the ISCT minimal criteria for MSCs might benefit from a re-evaluation for this particular detail.

Neu5Gc is an immunogenic xeno-carbohydrate that is not produced by humans due to the loss of hydroxylase activity of the human CMAH protein (Irie et al. 1998). MSCs cultured in presence of animal-derived material express this carbohydrate on their surface and intracellular proteins and it mediates immune responses against the cells (Heiskanen et al. 2007; Komoda et al. 2010). We demonstrate that PL1 and PL2 cultured cells are free of this animal-derived contaminant and thus not susceptible to antibodies against Neu5GC which are found in high titers in human serum.

The differentiation assays showed that MSCs cultured in PL1-medium are capable of tri-lineage differentiation. Although the tri-lineage differentiation is used as a criterion for MSCs in the research setting, it may not be relevant if the cells are utilized for immunosuppressive therapy in the clinic. The functional quality control tests for clinical products should be selected with a view to their intended use. In our study we tested the cells’ capacity to suppress T-cell proliferation and found effective suppression irrespective of culture condition.

The safety of the MSCs used for clinical purposes should be carefully assessed before administrating the cells to patients. Karyotype testing or other tests measuring genetic stability are often used. However, these tests may not be adequate to find small but deleterious abnormalities (Tarte et al. 2010). In our studies the karyotype was analysed using G-band staining and all tested batches had normal karyotype. Karyotype abnormalities have been noticed by others but usually at late passages and the cells enter into senescence without transformation irrespective of chromosomal alterations (Roselli et al. 2013; Tarte et al. 2010). The risk of tumor formation by MSCs harvested before senescence is, however, considered low (Capelli et al. 2011; Prockop et al. 2010; Tsai et al. 2011) and our protocol is based on a low amount of passaging when the cells are in a proliferative stage. Safety is further increased in our culture protocol by the omission of culture media antibiotics, and an unnecessary patient exposure to beta-lactam and aminoglycoside antibiotics can be avoided. Furthermore, in the absence of antibiotics, the risk of undetected bacterial contamination is reduced.

## Conclusions

We present a robust and reproducible clinically-compliant culture method for BM-MSCs based on PL, which enables high quantities of HLA-DR positive MSCs at a low passage number (p2) and with uniform quality. The cells were consistently HLA-DR positive when cultured in PL, but fulfill all other MSC criteria and suppress T-cell proliferation. The functional consequences of MSC HLA-DR expression need to be clarified in further studies. The animal serum-free, antibiotic-free, large-scale culture protocol can be directly transferred to a cleanroom environment for clinical-grade MSC manufacturing intended for allogeneic clinical use.

## Abbreviations

FBS: Fetal bovine serum
MSC: Mesenchymal stromal/stem cell
BM: Bone marrow
PL: Platelet lysate
PRP: Platelet rich plasma
CO_2_: Carbon dioxide
FRCBS: Finnish Red Cross Blood Service
MNC: Mononuclear cell
CFU-F: Colony forming unit-fibroblasts
PD: Population doubling
CD: Cluster of differentiation
HLA: Human leucocyte antigen
Neu5Gc: *N*-Glycolylneuraminic acid
PB: Peripheral blood
SD: Standard deviation
ANOVA: Analysis of variance
GMP: Good manufacturing practice
ISCT: International Society of Cell Therapy
MLR: Mixed lymphocyte reaction
